# U. S. Sanitary Commission

**Published:** 1861-09

**Authors:** 


U. S. SANITARY COMMISSION.—Drs. G. B. Wood, S. D. Gross, Robley Dunglison, and Wilson Jewell have been appointed associate members of this body from Philadelphia.

